# Feasibility pilot study of a Japanese teaching kitchen program

**DOI:** 10.3389/fpubh.2023.1258434

**Published:** 2023-12-07

**Authors:** Megu Y. Baden, Sarasa Kato, Akiko Niki, Tomoyuki Hara, Harutoshi Ozawa, Chisaki Ishibashi, Yoshiya Hosokawa, Yukari Fujita, Yuya Fujishima, Hitoshi Nishizawa, Junji Kozawa, Isao Muraki, Yusuke Furuya, Akio Yonekura, Tatsuro Shigyo, Taro Kawabe, Iichiro Shimomura, David M. Eisenberg

**Affiliations:** ^1^Department of Metabolic Medicine, Graduate School of Medicine, Osaka University, Suita, Japan; ^2^Department of Lifestyle Medicine, Graduate School of Medicine, Osaka University, Suita, Japan; ^3^Department of Diabetes Care Medicine, Graduate School of Medicine, Osaka University, Suita, Japan; ^4^Division of Public Health, Department of Social and Environmental Medicine, Graduate School of Medicine, Osaka University, Suita, Japan; ^5^Cancerscan, Inc, Tokyo, Japan; ^6^Kubara Honke Group Co., Ltd, Fukuoka, Japan; ^7^Department of Nutrition, Harvard T.H. Chan School of Public Health, Boston, MA, United States

**Keywords:** teaching kitchen, diet, obesity, lifestyle modification, behavior modification program

## Abstract

**Background:**

This pilot study examined the feasibility of a new lifestyle modification program involving a “Teaching Kitchen” in Japan. Our goal was to explore (1) feasibility of the program; (2) acceptability for class frequency (weekly vs. bi-weekly); and (3) changes in biometrics, dietary intakes, and lifestyle factors.

**Methods:**

A total of 24 employees with obesity in a Japanese company were recruited. Participants were randomly divided into two groups (weekly or bi-weekly group), each attending the program consisting of four two-hour classes (lectures on nutrition, exercise, mindfulness, and culinary instructions). Participants were observed for changes in dietary intakes, biometrics, and health related quality of life over the subsequent 3 months. We tested the between-group differences in changes using linear mixed-effect models.

**Results:**

The program completion rates were 83.3% in total (91.7% for weekly group and 75.0% for bi-weekly group). From baseline to post-intervention, significant decreases were observed in weight (*p* < 0.001), body mass index (*p* < 0.001), diastolic blood pressure (*p* = 0.03), body fat mass (*p* < 0.001), and dietary intakes in total fat (*p* = 0.03) and sodium (*p* = 0.008) among 17 participants who were available for measurements. Improvements in biometrics remained significant 1 month after the intervention (all *p* ≤ 0.03 in 14 participants). Participants' health related quality of life was significantly improved in bodily pain, general health, vitality, and mental component score (all *p* ≤ 0.047).

**Conclusions:**

The new Japanese Teaching Kitchen program is feasible with high program completion rates in Japanese office workers with obesity. While this was a small feasibility study, significant multiple improvements in dietary intakes, biometrics, and health related quality of life suggest that this line of inquiry warrants further exploration to address obesity and obesity-related diseases in Japan.

## 1 Introduction

The increase of obesity and obesity-related diseases is a severe global problem in both Western and Asian countries. Recently, a new lifestyle modification program referred to as a “Teaching kitchen” was developed and led by the researchers at the Harvard T.H. Chan School of Public Health (1, 2). Core components of Teaching Kitchen include: (1) nutrition lectures based on the latest scientific evidence; (2) hands-on culinary instruction to prepare healthy, delicious, and easy-to-prepare meals; (3) movement and exercise lectures; (4) mindfulness lectures; and (5) motivational interviewing and health coaching to sustain behavior change (2). Based on this concept, Teaching Kitchens are currently being held in a variety of settings, including hospitals, schools, and corporations, primarily in Western countries, with various program lengths and topics to suit the participants (2). Several previous studies on the efficacy of Teaching Kitchens reported that the curricula resulted in significant decreases in weight, waist circumference, blood pressure, and total cholesterol in the US individuals with obesity (3) and decreases in weight and HbA1c in Canadian patients with diabetes (4).

The traditional Japanese diet has been known to be healthy (5, 6). However, westernization of the Japanese diet and other lifestyle factors has led to a gradual increase in the Japanese population with obesity and obesity-related diseases since the 1960s (7). To modify lifestyles including diet, health guidance has recently been provided to Japanese individuals aged 40–74 who are enrolled in the National Health Insurance and have a risk of lifestyle related diseases (8). Similarly, companies provide their employees annual health checkups and lifestyle counseling to reduce their metabolic risk. In addition, many hospitals provide nutritional guidance for patients with diabetes by registered dietitians. Traditionally, most guidance is based on the Food Exchange Lists-Dietary Guidance for Persons with Diabetes (9), which provides a rough estimate of the amount of energy and nutrients contained in each food (10). Such strategy aims to restrict patients' calorie intake, restrain themselves from the consumption of snacks, and ensure macronutrient balance. However, the latest report from the Japanese National Institute of Health and Nutrition showed that the number of people with metabolic syndrome is continuing to increase in Japan (11). Therefore, more effective approaches are needed in addition to the current guidance to improve the diets and health outcomes of Japanese adults.

A previous US study reported that each 30 min decrease of food preparation time was associated with the increase in body mass index (BMI) of 0.5 between 1965 and 1995 (12). Another study in Japan with 9,143 men and 10,595 women aged more than 65 years showed that women had higher levels of cooking skills than men, and that both men and women with low level of cooking skills were more likely to have lower frequency of home cooking and unhealthy dietary behaviors than those with a high level of cooking (13). Several other studies have also consistently reported that there is a positive association between home cooking skills and diet quality (14–16). Therefore, it is considered that the acquisition of cooking skills at home is useful for motivating Japanese people to improve their diet. In addition, the National Health and Nutrition Survey in Japan showed that the obesity rate has increased rapidly among Japanese males in their 20s when most of them start living alone (17), implying that they do not have sufficient cooking skills to prepare healthy meals.

Therefore, if we can customize the US Teaching Kitchen curriculum developed by investigators from Harvard and the Teaching Kitchen Collaborative (18) for Japan and spread it into clinical practice and workplaces as a social implementation, this approach may serve as a powerful strategy to address, and potentially mitigate, the increasing obesity/obesity-related diseases rate and rising medical costs in Japan. To meet such critical needs, in the current study we developed a new Japanese Teaching Kitchen program by building the professional team with Japanese physicians specialized in diabetology and metabolism, public health specialists, registered dietitians, culinary specialists (a.k.a., cooks), and companies that specialized in behavior modification (Cancerscan, Inc) and the production of Japanese traditional foods (Kubara Honke Group Co., Ltd). Our goal was (1) to examine the feasibility of a potentially replicable Teaching Kitchen program/curriculum as applied to a Japanese population of worksite employees; (2) to determine if a frequency of classes of once per week vs. once per 2 weeks was accepted by participants over four classes; and 3) to investigate and report on changes in relevant lifestyle factors, biometrics, and health related quality of life (HR-QoL), before and after the program intervention.

## 2 Materials and methods

### 2.1 Recruitment

The participants of the current study are the employees in the NISSIN Foods Holdings Co., Ltd (Tokyo, Japan) working at that company's main office (Shinjuku, Tokyo) and at the company's research center (Hachioji, Tokyo). The NISSIN Foods Holdings Co., Ltd had no conflict of interest to this study. Subject recruitment criteria included: those with the ages of 20 to 74 and with BMI≥25 and/or waist circumstance ≥85 cm for men and ≥90 cm for women (consistent with the diagnostic criteria of metabolic syndrome in Japan). The exclusion criteria were those who needed special dietary therapy such as protein restriction and/or severe food allergy, those with severe hypertension (systolic blood pressure ≥160 mmHg and diastolic blood pressure ≥110 mmHg), those with severe hypertriglyceridemia (≥1000 mg/dl), those who required exercise restriction (e.g., those with heart failure and/or unstable angina), those who were pregnant, planning to become pregnant, or were breastfeeding, and those who the researchers judged difficult to participate in the program (e.g., those who were going to be absent due to a long trip). Based on the most recent health checkup data, employees who met the criteria were invited to participate in this study via e-mails from the staffs of the company. After signing the informed consent, participants were randomly divided into two groups: the weekly group, who attended four weekly classes; and the bi-weekly group, who attended four classes every other week. We employed a stratified randomization method which controlled for age, sex, BMI, and working place (Supplementary Table 1). On stratification, the participants were not asked their preference regarding the frequency of classes.

The current study was approved by the Institutional Ethics Review Board of Osaka University Hospital (approved number 21335) and informed consent was obtained from all participants.

### 2.2 Interventions

Based on the concept of a Teaching Kitchen which includes hands-on culinary instruction and lifestyle guidance based on the scientific evidence (2), the Japanese Teaching Kitchen program has been developed by building the team with Japanese physicians specialized in diabetology and metabolism, public health specialists, registered dietitians, culinary specialists, and companies specialized in behavior modification (Cancerscan, Inc, the Japanese start-up company providing preventive healthcare service to local governments) and the production of Japanese traditional foods (Kubara Honke Group Co., Ltd). In developing the Japanese Teaching Kitchen program, all healthy recipes created for the program were based on the healthy Japanese diet, using foods that are available in Japan on a daily basis and using cooking methods commonly used in Japan (e.g., boiling, steaming, and baking). In addition, since the Japanese diet has a higher sodium intake than that of Western countries (19), the Japanese Teaching Kitchen program particularly focused on how to reduce salt intake. Specifically, we made use of *dashi*, a fish soup stock that contains a lot of umami, which is traditionally used in Japan, as well as spices and seasonings to reduce salt intake while maintaining food satisfaction.

**Figure 1:**
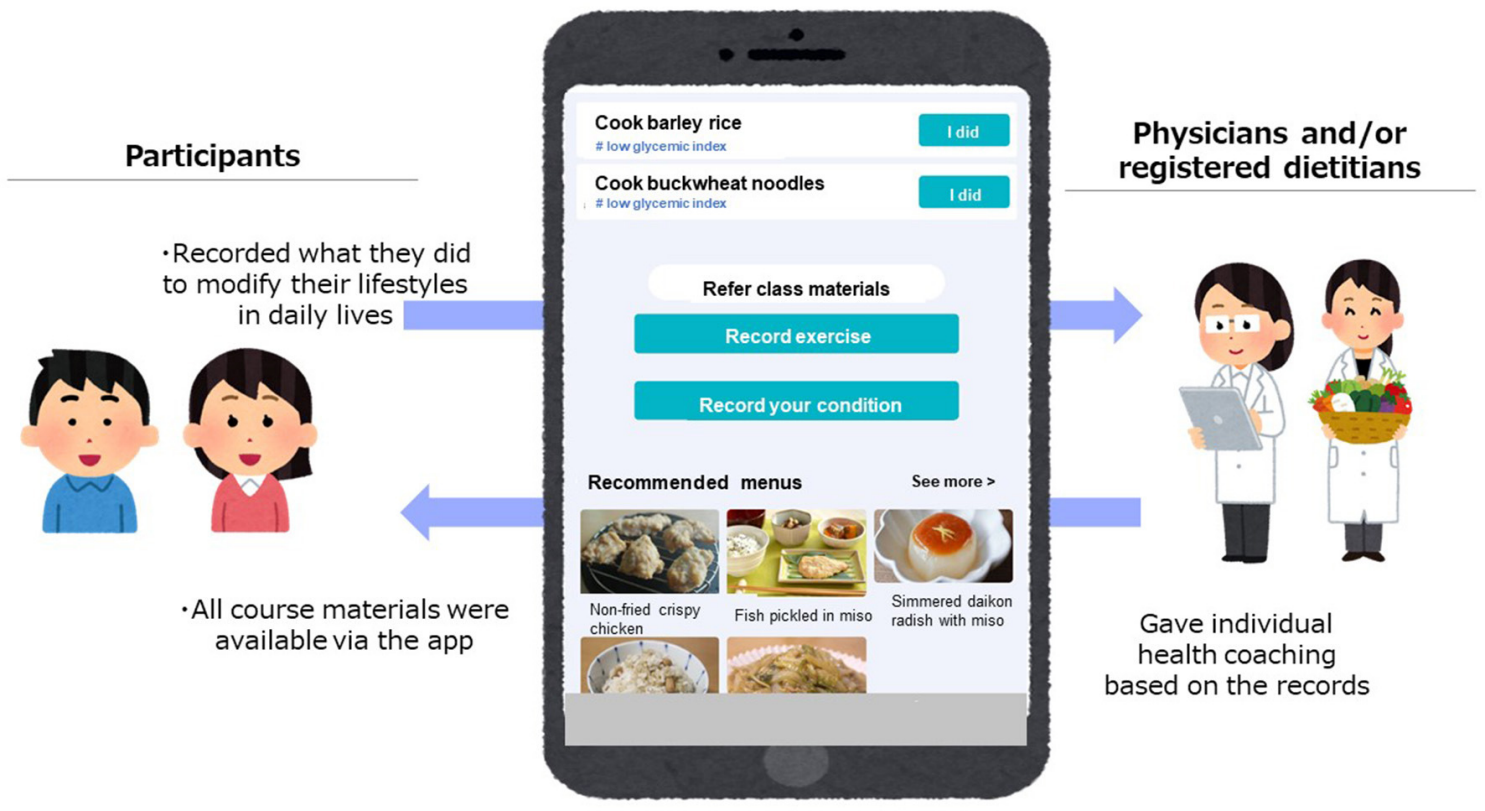
The overview of the web application.

The intervention included four 2-hour face-to-face classes (one month for four weekly classes and 2 months for four biweekly classes). All classes took place at the kitchens owned by ABC Cooking Studio Co. Ltd (Tokyo, Japan) and were facilitated by the researchers (physicians, registered dietitians, and chefs). The example of timetable for the face-to-face class was shown in Supplementary Table 2. During the classes, participants attended lectures on nutrition, physical activity, and mindfulness, as well as hands-on culinary lessons working in assigned teams of 4–6 people to cook the healthy meals with chefs and then eat the foods they prepared by themselves. Each class had a specific topic (how to lower calorie intake, how to lower salt intake, how to lower glycemic index, and healthy party menus including appetizers and snacks) and participants learned the nutrition evidence and practical skills related to the topic in lectures and cooking experience. The participants also used a web application (app) designed for this study by the researchers and Cancerscan, Inc during the intervention. The app had mainly two functions. First, participants could access all class materials including the recipes and recommended menus that helped participants to cook healthy dishes at home and to choose healthy menus when they ate out (Figure 1). Second, participants could record what they did in their daily lives to modify their lifestyles (e.g., cooked healthy dishes they learned, chose healthy menus, did exercise) by posting the text and/or pictures via the app and researchers (physicians and/or registered dietitians) replied with encouraging advice as an individual health coaching (Figure 1).

### 2.3 Outcome assessments

**Figure 2:**
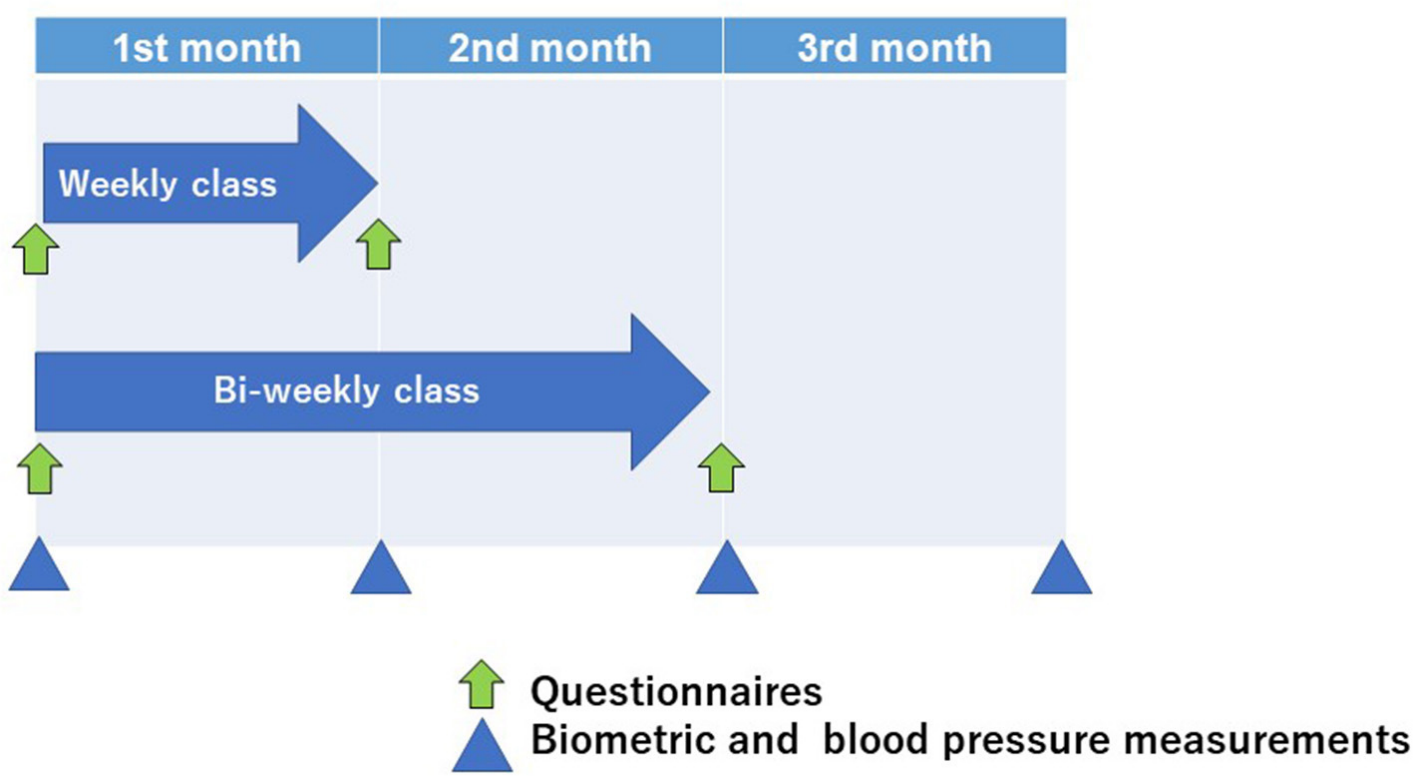
The timeline of assessments.

Feasibility was assessed through recruitment, program completion rate, and satisfaction with classes as a primary outcome. We assessed acceptability for class frequency and changes in biometrics, dietary intakes, and lifestyle factors as secondary outcomes. The timeline of assessments was shown in Figure 2 and all instruments used in this study were listed in Supplementary Table 3. Biometric outcomes and blood pressure were assessed four times: at baseline, 1 month later, 2 months later, and 3 months later. Body weight and the amounts of body fat mass and lean mass were evaluated using body composition analyzer (MC-780A-N, TANITA Co. Ltd, Tokyo, Japan). Weights were measured to one decimal place, with participants wearing light clothing, shoes removed, and 0.5 kg subtracted for the weight of clothing. Blood pressure was measured in the sitting position by the researchers (physician) using a digital sphygmomanometer (TERUMO, Tokyo, Japan). Changes in biometric and blood pressure at baseline, end of the intervention, and 1 month after the intervention were assessed using the baseline values, values at the end of the program (four weeks later for the weekly group and 8 weeks later for the bi-weekly group), and values at the end of the one-month follow-up (8 weeks later for the weekly group and 12 weeks later for the bi-weekly group). Several validated questionnaires were used to assess the difference of participants' diets, physical activity, sleep difficulty, and HR-QoL between baseline and at the end of the program (four weeks later for the weekly group and 8 weeks later for the bi-weekly group). Dietary intakes were examined using the self-administered long-food frequency questionnaire (FFQ) developed for Japanese individuals (20). Food intake was calculated by multiplying the frequency of consumption (never, 1–3 times/month, 1–2 times/week, 3–4 times/week, 5–6 times/week, once/day, 1–2 times/day, 4–6 times/day, 7+ times/day) by relative portion size (small, medium, and large), and participants were asked to report dietary intake in the previous year for the baseline and dietary intake during the intervention for the post-intervention assessment. Obesity-related eating behavior was quantified using the questionnaire of the guideline for obesity issued by the Japan Society for the Study of Obesity to identify the problems in various eating behaviors, following previous studies (21–23). This questionnaire consists of 55-item questions of seven major scales as follows: 1. Recognition for weight and constitution (e.g., “Do you think it is easier for you to gain weight than others?”, “Do you think you gain weight because of less exercise?”), 2. External eating behavior (e.g., “If food smells and looks good, do you eat more than usual?”, “If you walk past the supermarket, do you have the desire to buy something delicious?”, “If you see others eating, do you also have the desire to eat?”), 3. Emotional eating behavior (e.g., “Do you have the desire to eat when you are irritated?”, “Do you have a desire to eat when you have nothing to do?”), 4. Sense of hunger (e.g., “Do you get irritated when you feel hungry?”, “Do you often regret because you have eaten a lot of food?”), 5. Eating style (e.g., “Do you eat fast?”, “Are you known to eat a lot of food?”), 6. Food preference (e.g., “Do you often eat snack bread?”, “Do you like meat?”, “Do you like noodles?”), 7. Regularity of eating habits (e.g., “Is your dinner time too late at night?”, “Do you gain body weight during holidays?”). All items were rated on a four-point scale ranging from 1 (seldom) to 4 (very often) and higher scores mean poorer eating behaviors. Physical activity was assessed using International Physical Activity Questionnaire (IPAQ). Sleep difficulty was quantified using Athens Insomnia Scale. HR-QoL was evaluated using the medical Outcomes Study 36-Item Short-Form Health Survey (SF-36), version 2 (24). In addition, participants put smart bands (Mi Band 6, Xiaomi, China) to assess their physical activity and sleep duration during the study period. Participants' cooking frequency (times/week) and the percentage of skipping meals more than once a week were asked by the self-reported questionnaire before and after the intervention. Further, after the intervention, participants reported their satisfaction for each content of the face-to-face classes (nutrition lecture, cooking, exercise lecture, and mindfulness lecture) by choosing the numbers from 0 to 10 (0; not satisfied and 10: most satisfied) and whether they felt their diet have improved or not.

### 2.4 Statistical analyses

The program completion rate was determined by the percentage of participants who completed the study. To compare the program completion rate, satisfaction rating, and characteristics of participants between weekly and bi-weekly groups, *t*-tests were used for continuous variables and Fisher's exact test was used for categorical variables. We confirmed the normality of each continuous variable by drawing a probability plot and found that age, alcohol intake, and satisfaction rating for the nutrition class were not in the normal distribution. For these three variables, we tested the between-group difference by the Willcoxon rank test. The changes in biometrics, HR-QoL, and lifestyles between before and after the program intervention were examined by using linear mixed effect models. Changes in outcomes from baseline to post-intervention (when the program finished) were examined in the participants who finished biometric measurements both at baseline and after the intervention. The changes from baseline to 1 month after the program were examined in the participants who finished measurements three times (baseline, when they finished the program, and after the 1 month follow up). All All analyses were conducted using SAS version 9.4 (SAS Institute, Inc, Cary, NC) with statistical significance defined as two-sided *p* < 0.05.

## 3 Results

### 3.1 Program completion rates and baseline characteristics of participants in weekly and bi-weekly groups

**Figure 3:**
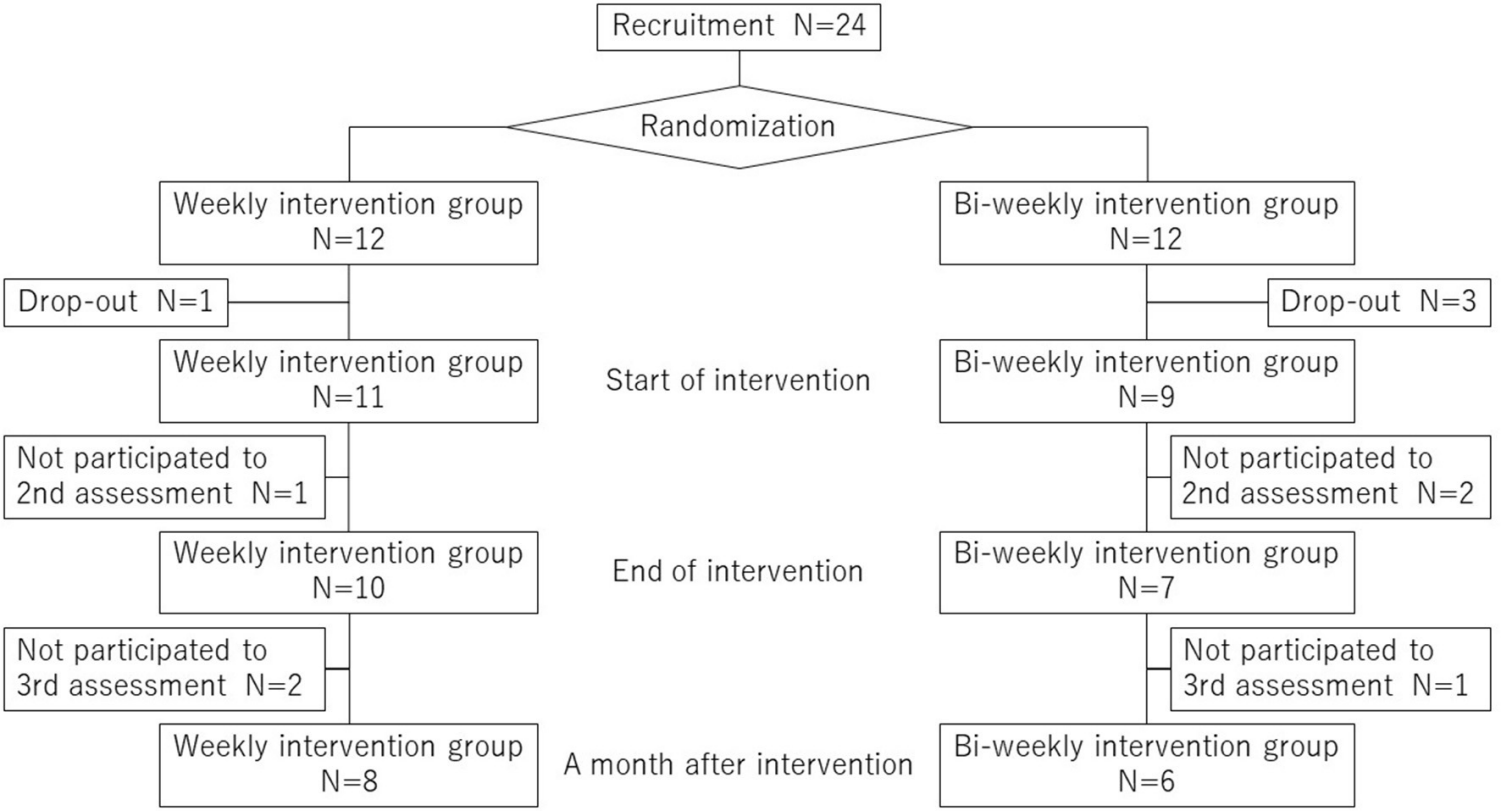
A flow diagram with the recruitment, randomization, intervention, and follow-up.

Approximately 300 employees who met the criteria were sent e-mails for recruitment into the study, and 24 participants were recruited within 3 weeks. By inviting employees who met the criteria, the recruited participants were predominantly male (22 males and 2 females, Supplementary Table 1). A flow diagram with the recruitment, randomization, intervention, and follow-up was shown in Figure 3. Baseline characteristics of the participants and program completion rates in weekly and bi-weekly groups are summarized in Table 1. Within the 12 participants in each group, one participant in the weekly group and three participants in the bi-weekly group dropped out from the program. All participants who quit the program expressed their withdrawal before they attended the first class. Reasons for their dropping out were one for family matter and three relating to their work schedules. Consequently, the program completion rates were 83.3% in total, with 91.7% in the weekly group and 75.0% in the bi-weekly group. When participants were asked for their ratings with respect to the content of the classes, with a range from 0 (not satisfied) to 10 (most satisfied), their approval scores (mean±SD) in all participants were 8.6 ± 1.7 for the nutrition lectures, 7.5 ± 1.8 for the cooking classes, 7.2 ± 2.0 for the exercise lectures, and 6.2 ± 2.1 for the mindfulness lectures. When we compared the satisfaction ratings for each content of the classes in the weekly and bi-weekly groups, there were no significant differences between the two groups, except for the mindfulness lecture (Table 2).

**Table 1:** Baseline characteristics of participants in weekly group and bi-weekly group.

	**Weekly group**	**Bi-weekly group**	***p***
*N*	11	9	
Age, year	38 [35, 52]	39 [38, 43]	0.30
Sex (male/female)	10/1	8/1	1.00
Weight, kg	80.3 ± 10.4	81.6 ± 13.5	0.81
BMI, kg/m^2^	27.2 ± 2.3	27.4 ± 4.1	0.93
Body fat mass, kg	22.1 ± 6.6	24.9 ± 8.3	0.42
Lean mass, kg	55.2 ± 7.7	53.8 ± 7.5	0.69
Systolic blood pressure, mmHg	131.7 ± 10.0	137.4 ± 16.5	0.35
Diastolic blood pressure, mmHg	86.7 ± 7.0	87.3 ± 8.9	0.87
Physical activity, MET-h/week	23.5 ± 15.3	23.4 ± 17.9	0.98
Athens Insomnia Scale	4.5 ± 1.8	7.4 ± 4.4	0.08
Living alone, %	18.2	44.4	0.34
Office (main office/research center), %	81.8/18.2	88.9/11.1	1.00
Smoking (current, past, none), %			0.25
Current	0	11.1	
Past	54.6	22.2	
None	45.5	66.7	
Alcohol intake, g/day^*^	2.1 [0.5, 11.5]	4.9 [0.5, 16.9]	0.32
Hypertension, %	36.4	11.1	0.32
Hyperlipidemia, %	18.2	11.1	1.00
Diabetes, %	9.1	0	1.00
Sleep Apnea Syndrome, %	0	22.2	0.19
Program completion rate, %	91.7	75.0	0.59

Values are means ± standard deviations (SDs) for continuous variables and percentages for categorical variables. The differences between weekly and bi-weekly groups were tested using t-tests for continuous variables other than age and alcohol intake, the Willcoxon rank test for age and alcohol intake, and Fisher's exact test for categorical variables. p <0.05 indicates statistically significant. BMI, body mass index; MET, metabolic equivalent; n, the number of participants. ^*^ Values are median [interquartile range] and tested by using the Wilcoxon rank test.

**Table 2:** Participants' satisfaction ratings of each content of classes in weekly group and bi-weekly group.

	**Weekly group**	**Bi-weekly group**	***P***
*n* ^a^	10	6	
Nutrition lecture	9.5 [8, 10]	8.5 [7, 10]	0.32
Cooking class	7.1 ± 1.7	8.2 ± 1.7	0.25
Exercise lecture	6.9 ± 2.0	7.7 ± 2.1	0.48
Mindfulness lecture	5.3 ± 2.1	7.7 ± 1.2	0.03

Values are means ± standard deviations (SDs) or median [interquartile range]. The differences between weekly and bi-weekly groups were tested using the Willcoxon rank test for the Nutrition lecture and t-tests for the others. p <0.05 indicates statistically significant. ^***a***^n = 16 instead of 17 because of missing values.

### 3.2 Changes in biometrics from baseline to post-intervention

From baseline to post-intervention (four weeks for the weekly group and 8 weeks for bi-weekly group), significant decreases were observed in weight, BMI, systolic blood pressure, diastolic blood pressure, and body fat mass (Table 3). On the other hand, the lean mass remained unchanged (Table 3). These significant biometric improvements were maintained by participants 1 month after the intervention (Supplementary Table 4). There were no differences in biometric improvements between the weekly and the bi-weekly groups (Table 3 and Supplementary Table 4). Percent changes in biometrics were shown in Supplementary Tables 5, 6.

**Table 3:** Changes in biometrics at baseline and post-intervention.

	**Baseline**	**Post-intervention**	**Mean change**	***p***	**p for interaction**
Weight, kg					
Weekly (*n =* 10)	81.2 ± 3.3	80.0 ± 3.4	−1.2 ± 0.3	<0.001	
Bi-weekly (*n =* 7)	84.1 ± 5.5	82.4 ± 5.0	−1.8 ± 0.8	0.04	
Total (*n =* 17^a^)	82.4 ± 2.9	80.9 ± 2.8	−1.4 ± 0.4	<0.001	0.42
BMI, kg/m^2^					
Weekly (*n =* 10)	27.3 ± 0.8	26.9 ± 0.8	−0.4 ± 0.1	<0.001	
Bi-weekly (*n =* 7)	27.6 ± 1.7	27.0 ± 1.5	−0.6 ± 0.3	0.04	
Total (*n =* 17)	27.4 ± 0.8	26.9 ± 0.8	−0.5 ± 0.1	<0.001	0.45
SBP, mmHg					
Weekly (*n =* 10)	134 ± 3	132 ± 6	−2 ± 4	0.60	
Bi-weekly (*n =* 7)	140 ± 7	127 ± 4	−13 ± 5	0.02	
Total (*n =* 17)	136 ± 4	130 ± 4	−7 ± 2	0.05	0.09
DBP, mmHg					
Weekly (*n =* 10)	88 ± 2	83 ± 3	−5 ± 2	0.03	
Bi-weekly (*n =* 7)	87 ± 4	84 ± 4	−2 ± 3	0.46	
Total (*n =* 17)	87 ± 2	83 ± 2	−4 ± 2	0.03	0.49
Body fat mass, kg					
Weekly (*n =* 10)	22.5 ± 2.2	21.5 ± 2.2	−1.0 ± 0.2	<0.001	
Bi-weekly (*n =* 7)	25.0 ± 3.4	22.9 ± 2.7	−2.1 ± 0.8	0.03	
Total (*n =* 17)	23.5 ± 1.8	22.1 ± 1.7	−1.5 ± 0.4	<0.001	0.15
Lean mass, kg					
Weekly (*n =* 10)	55.6 ± 2.5	55.5 ± 2.5	−0.2 ± 0.3	0.63	
Bi-weekly (*n =* 7)	56.1 ± 2.2	56.3 ± 2.2	0.3 ± 0.7	0.71	
Total (*n =* 17)	55.8 ± 1.7	55.8 ± 1.7	0.0 ± 0.4	0.96	0.54

Values are means ± standard errors (SEs). ^***a***^n = 17 instead of 20 because measurements were not available for three participants because of work commitments. The baseline and post-intervention differences were tested using linear mixed effect models. p <0.05 indicates statistically significant. BMI, body mass index; SBP, systolic blood pressure; DBP, diastolic blood pressure.

### 3.3 Changes in dietary habits

**Figure 4:**
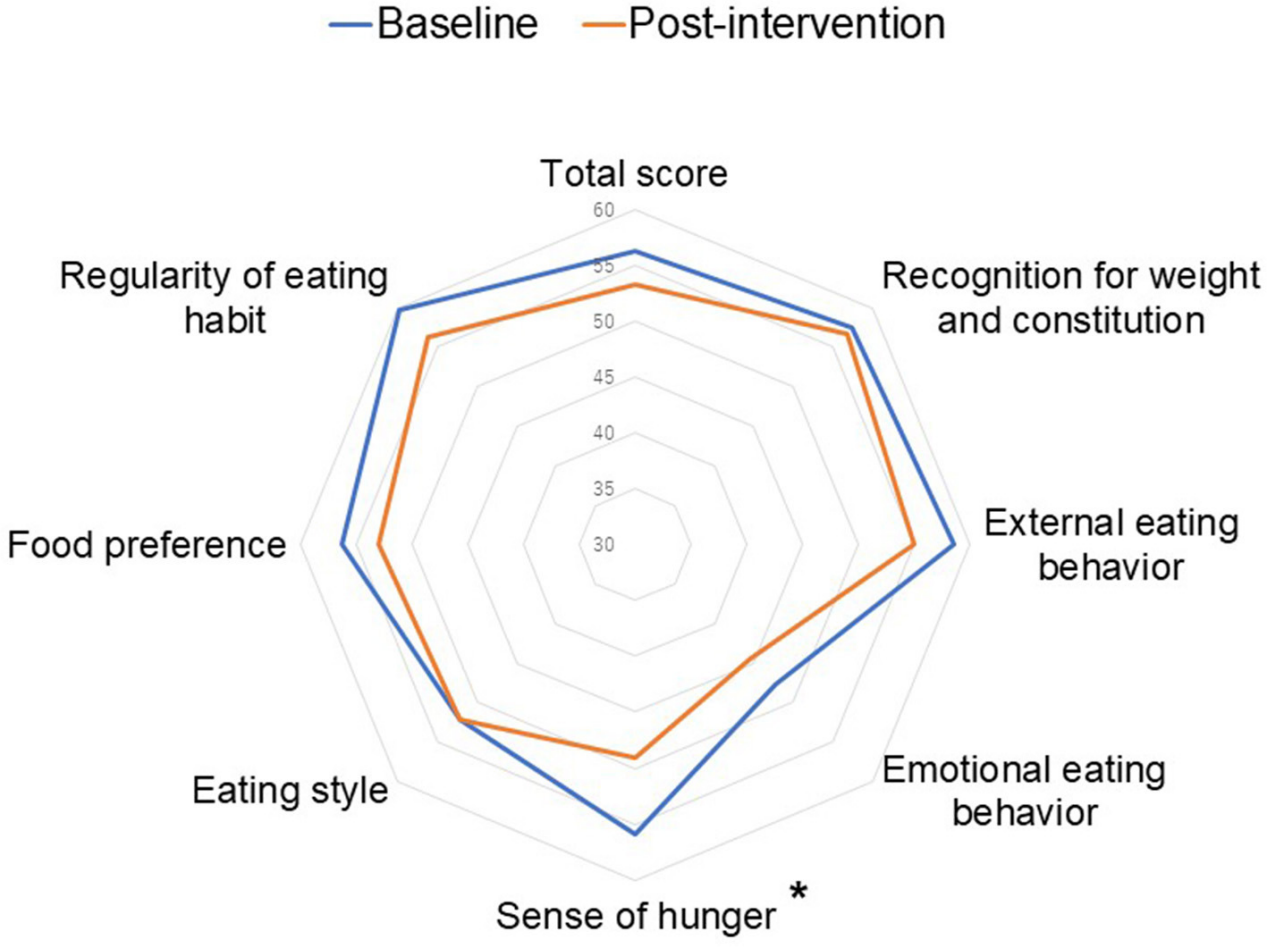
Comparisons of obesity-related eating behaviors between baseline and post-intervention (*n*^a^ = 16). The baseline and post-intervention differences were tested using linear mixed effect models. ^a^*n* = 16 instead of 17 because of the missing value. *indicates *p* < 0.05.

Compared to the baseline, in total, there were significant decreases in total fat intake and sodium intake (*p* = 0.03 and 0.008, respectively, Table 4). When we compared the changes in dietary intakes between the weekly and the bi-weekly groups, the tendencies of changes were almost identical except for fruit intake (p for interaction=0.04). Percent changes in dietary intakes were shown in Supplementary Table 7. As shown in Figure 4, participants' obesity-related eating behaviors tended to improve from baseline to post-intervention in total score and significantly improved in the sense of hunger (*p* = 0.04, the decreased score means the improvement of eating behaviors). In addition, based on the self-reported questionnaire, the percentage of skipping breakfast tended to decrease from 41.2 to 17.6% (*p* = 0.23) whereas there were no differences in skipping lunch (5.9 to 5.9%, *p* = 1.00) and dinner (0 to 0%, *p* = 1.00). For cooking frequency, we observed no differences from baseline until the end of the intervention (from 6.6 times/week to 6.3 times/week, *p* = 0.62).

**Table 4:** Changes in energy, nutrient and food intakes at baseline and post-intervention.

	**Baseline**	**Post-intervention**	**Mean change**	***p***	**P for interaction**
Total energy intake, kcal					
Weekly (*n =* 10)	2176 ± 71	2133 ± 63	−43 ± 49	0.39	
Bi-weekly (*n =* 7)	2247 ± 55	2162 ± 40	−85 ± 28	0.01	
Total (*n =* 17)	2205 ± 47	2145 ± 40	−60 ± 30	0.06	0.52
Protein, g					
Weekly (*n =* 10)	86.3 ± 1.8	85.2 ± 1.9	−1.2 ± 1.2	0.33	
Bi-weekly (*n =* 7)	88.9 ± 1.8	86.6 ± 0.9	−2.3 ± 1.1	0.07	
Total (*n =* 17)	87.4 ± 1.2	85.8 ± 1.1	−1.6 ± 0.8	0.05	0.50
Total fat, g					
Weekly (*n =* 10)	64.0 ± 1.1	63.1 ± 1.1	−1.0 ± 0.9	0.3	
Bi-weekly (*n =* 7)	65.9 ± 0.8	63.8 ± 0.9	−2.1 ± 0.7	0.01	
Total (*n =* 17)	64.8 ± 0.7	63.4 ± 0.7	−1.4 ± 0.6	0.03	0.38
Carbohydrate, g					
Weekly (*n =* 10)	286.6 ± 14.8	283.2 ± 12.8	−3.3 ± 10.0	0.75	
Bi-weekly (*n =* 7)	288.3 ± 11.3	282.0 ± 6.1	−6.3 ± 6.4	0.34	
Total (*n =* 17)	287.3 ± 9.6	282.7 ± 7.8	−4.5 ± 6.3	0.48	0.82
Total dietary fiber, g					
Weekly (*n =* 10)	17.9 ± 0.5	17.7 ± 0.5	−0.2 ± 0.4	0.66	
Bi-weekly (*n =* 7)	19.2 ± 1.2	18.8 ± 0.9	−0.4 ± 0.6	0.51	
Total (*n =* 17)	18.4 ± 0.6	18.2 ± 0.5	−0.3 ± 0.3	0.41	0.73
Sodium, g					
Weekly (*n =* 10)	10.7 ± 0.4	10.3 ± 0.4	−0.4 ± 0.3	0.15	
Bi-weekly (*n =* 7)	11.7 ± 0.7	10.8 ± 0.4	−0.9 ± 0.3	0.02	
Total (*n =* 17)	11.1 ± 0.4	10.5 ± 0.3	−0.6 ± 0.2	0.008	0.24
Vegetables, g					
Weekly (*n =* 10)	338.6 ± 9.1	349.5 ± 15.5	10.9 ± 16.1	0.51	
Bi-weekly (*n =* 7)	390.7 ± 35.2	412.0 ± 24.6	21.3 ± 30.9	0.5	
Total (*n =* 17)	360.1 ± 16.1	375.2 ± 15.2	15.2 ± 15.3	0.33	0.75
Fruits, g					
Weekly (*n =* 10)	131.9 ± 4.8	129.3 ± 4.7	−2.6 ± 1.4	0.09	
Bi-weekly (*n =* 7)	140.7 ± 14.6	150.6 ± 20.5	9.9 ± 6.8	0.17	
Total (*n =* 17)	135.5 ± 6.4	138.1 ± 8.9	2.5 ± 3.2	0.43	0.04
Fish and shellfish, g					
Weekly (*n =* 10)	101.1 ± 2.2	101.9 ± 3.2	0.8 ± 2.3	0.73	
Bi-weekly (*n =* 7)	106.1 ± 5.1	105.3 ± 3.7	−0.9 ± 6.2	0.89	
Total (*n =* 17)	103.2 ± 2.5	103.3 ± 2.4	0.1 ± 2.8	0.97	0.78
Meats, g					
Weekly (*n =* 10)	73.8 ± 2.6	72.1 ± 4.8	−1.7 ± 5.0	0.74	
Bi-weekly (*n =* 7)	79.3 ± 5.0	69.1 ± 4.9	−10.1 ± 3.8	0.02	
Total (*n =* 17)	76.1 ± 2.5	70.9 ± 3.4	−5.2 ± 3.4	0.14	0.22
Eggs, g					
Weekly (*n =* 10)	35.5 ± 0.4	34.9 ± 0.7	−0.6 ± 0.7	0.38	
Bi-weekly (*n =* 7)	37.3 ± 1.7	36.7 ± 0.6	−0.6 ± 1.3	0.66	
Total (*n =* 17)	36.2 ± 0.7	35.6 ± 0.5	−0.6 ± 0.6	0.36	0.98
Dairy, g					
Weekly (*n =* 10)	194.1 ± 42.7	162.4 ± 22.2	−31.7 ± 43.7	0.48	
Bi-weekly (*n =* 7)	195.7 ± 38.8	187.4 ± 17.1	−8.3 ± 34.2	0.81	
Total (*n =* 17)	194.8 ± 28.9	172.7 ± 14.8	−22.1 ± 28.7	0.45	0.70
Alcoholic beverages, g					
Weekly (*n =* 10)	8.9 ± 4.4	4.3 ± 1.7	−4.7 ± 3.6	0.21	
Bi-weekly (*n =* 7)	11.2 ± 4.9	9.6 ± 4.2	−1.6 ± 0.9	0.11	
Total (*n =* 17)	9.9 ± 3.2	6.5 ± 2.0	−3.4 ± 2.2	0.12	0.64
Confectionaries, g					
Weekly (*n =* 10)	24.5 ± 5.2	19.2 ± 4.7	−5.3 ± 3.6	0.16	
Bi-weekly (*n =* 7)	30.6 ± 7.3	21.7 ± 10.2	−8.9 ± 7.1	0.24	
Total (*n =* 17)	27.0 ± 4.2	20.2 ± 4.8	−6.8 ± 3.5	0.06	0.63

Values are means ± standard errors (SEs). The baseline and post-intervention differences were tested using linear mixed effect models. p <0.05 indicates statistically significant.

### 3.4 Changes in physical activity

As shown in Table 5, the amount of physical activity assessed using the IPAQ tended to increase from 21.6 ± 3.7 MET-h/week at baseline to 25.6 ± 4.3 MET-h/week in the post-intervention assessment (*p* = 0.20). The mean changes of physical activity from baseline to post-intervention were 4.6 ± 3.3 MET-h/week in weekly group and 2.8 ± 7.2 MET-h/week in bi-weekly group (p for interaction=0.79). Percent changes in physical activity were shown in Supplementary Table 8. We also assessed the amount of physical activity including dairy activities during the intervention by using smart bands. As shown in Supplementary Table 9, the amount of physical activity assessed by the smart bands were 29.0 ± 4.4 MET-h/week in total, 36.4 ± 6.7 MET-h/week in weekly group, and 20.5 ± 3.1 MET-h/week in bi-weekly group (p for interaction = 0.07).

**Table 5:** Changes in physical activity and Athens insomnia scale at baseline and post-intervention.

	**Baseline**	**Post-intervention**	**Mean change**	***p***	**p for interaction**
Physical activity, MET-h/week					
Weekly (*n =* 10)	22.8 ± 5.0	27.4 ± 6.1	4.6 ± 3.3	0.17	
Bi-weekly (*n =* 5)	19.2 ± 5.1	22.0 ± 5.1	2.8 ± 7.2	0.71	
Total (*n =* 15)	21.6 ± 3.7	25.6 ± 4.3	4.0 ± 3.1	0.20	0.79
Athens Insomnia Scale					
Weekly (*n =* 10)	4.4 ± 0.6	3.4 ± 1.1	−1.0 ± 0.9	0.30	
Bi-weekly (*n =* 6)	8.4 ± 1.9	7.0 ± 2.2	−1.2 ± 1.6	0.48	
Total (*n =* 16)	5.8 ± 0.9	4.8 ± 1.1	−1.1 ± 0.8	0.19	0.92

Values are means ± standard errors (SEs). Physical activities were assessed using the international physical activity questionnaire (IPAQ). n = 15 for physical activity and 16 for Athens insomnia scale instead of 17 because of missing values. The baseline and post-intervention differences were tested using linear mixed effect models. p <0.05 indicates statistically significant. MET, metabolic equivalent.

### 3.5 Changes in sleeping habit

From the baseline to post-intervention, the Athens Insomnia Scale changed from 5.8 ± 0.9 to 4.8 ± 1.1 (*p* = 0.19, Table 5). Percent changes in the Athens Insomnia Scale were shown in Supplementary Table 8. The sleep duration during the intervention assessed by using smart bands was 6.3 ± 0.3 hour in all participants, 6.7 ± 0.3 hour in weekly group, and 5.9 ± 0.4 hour in bi-weekly group (p for interaction = 0.20, Supplementary Table 9).

### 3.6 Changes in health-related quality of life

Changes in the summary scores and each domain score of HR-QoL were shown in Supplementary Table 10. From the baseline to post-intervention, there was a significant improvement in mental component score (*p* = 0.01). For the eight domains of HR-QoL, bodily pain, general health, and vitality were significantly improved (*p* = 0.03, 0.006, and *p* = 0.047, respectively). Percent changes in HR-QoL scores were shown in Supplementary Table 11. There was no difference in the improvements of summary scores and domains of HR-QoL between weekly and bi-weekly group (all p for interaction ≧ 0.33).

## 4 Discussion

To our knowledge, the current study is the first feasibility study of a Japanese Teaching Kitchen program involving Japanese employees with obesity. Our results suggest that the Japanese Teaching Kitchen program is feasible in this workplace with high program completion rates (91.7% for the weekly group and 75.0% for the bi-weekly group); and, that this program led to improvements in participants' biometrics, lifestyle behaviors, and HR-QoL.

In line with Teaching Kitchen programs originally developed in the US (1, 2), our Japanese Teaching Kitchen program offers a comprehensive approach to modify participants' diet, exercise, and mindfulness, incorporating both didactic and experimental learning. In face-to-face classes, lecturers introduced various recent scientific evidence on nutrition, physical activity, and sleeping habits to deepen participants' understanding of healthy lifestyles. In addition, lecturers offered simple and practical measures to modify their diet and other lifestyle factors. For example, the participants learned to better assess the differences in calories in each type of meat (e.g., learned the calories of loin vs. filet of beef) and learned the difference in calories when employing different cooking methods (e.g., learned the calories of dishes made when frying vs. grilling ingredients) that enabled them to choose healthier foods and dishes to lower calorie intakes. In addition, participants learned about aerobic and resistance exercises that are appropriate for them in the exercise lectures and breathing techniques for relaxation in the mindfulness lecture. Following these lectures, participants made dishes using these techniques, and enjoyed the simplicity and deliciousness of what they cooked. This type of educational approach integrating lectures and culinary experience effectively motivated participants to incorporate what they learned into their daily lives. It is noteworthy that this approach enabled participants to improve their dietary intakes without strict prohibitions and concomitant feelings of perceived deprivations which often accompany usual diet related therapies, the majority of which are restrictive in nature.

One of the originalities of the Japanese Teaching Kitchen program is that the program made use of the effectiveness of *dashi*, the fish soup stock traditionally used in Japan, to increase food satisfaction of the participants. *Dashi* contains a lot of umami, which is one of the basic tastes (25). Umami stimulates the amygdala in the brain to increase food satisfaction (26) and suppresses overeating (27). In addition, *dashi* contains multiple umami components such as glutamic acid (mainly from kelp), inosinic acid (bonito flakes), and guanylic acid (shiitake mushrooms), and strong umami stimulation is obtained by synergistic effects of multiple umami components (28, 29). Actually, in this study we observed the significant decrease in sodium intake by Japanese Teaching Kitchen program, which is in line with a previous study reported that consumption of umami-rich diets decreased salt intake (30). In addition, the improvements in other dietary intakes as well as sodium intake and obesity-related eating behaviors shown in this study indicate that the program contents were effective for the population.

Another original approach in the Japanese Teaching Kitchen program is that we have developed a web app and used it in conjunction with the face-to-face classes. The app enabled participants to access class materials, record what they did to improve their lifestyles, and receive message from physicians and dietitians based on their records. In the current study the participants could only use the app during the intervention (four weeks for weekly group and eight weeks for bi-weekly group) to assess the feasibility of this app-based approach. As the next step, we will modify the app to maintain the efficacy of the Japanese Teaching Kitchen program even after the intervention finishes and examine its' effectiveness and potential for improvement in another study.

In this study, we randomly divided participants into weekly and bi-weekly groups independent from their preferences to see which is more feasible and effective. The weekly group showed higher program completion rates than the bi-weekly group and may be more acceptable to Japanese adults. There were no significant differences in satisfaction with class contents between the two groups, except for the mindfulness lecture. This difference may be influenced by the small number of participants who completed the satisfaction questionnaire (*n* = 6) in the bi-weekly group. It is worth noting that the completion rates and patients' satisfaction with the program in total were relatively high regardless of their preferences for weekly vs bi-weekly classes. However, at the same time, the participants' preferences may have affected the completion rates and satisfaction. We will assess these factors in another intervention study in which designing all participants will take classes at the same frequency.

The current study is the first feasibility study of Japanese Teaching Kitchen program and showed the feasibility and effectiveness of the program in various aspects. However, there are several limitations that should be noted. First, all participants were employees of one Japanese company (NISSIN Foods Holdings). Although the company had no input into the design of the study, or analysis of the data collected, their cooperation in recruiting participants (sending emails for recruitment and allowing participants to take the Teaching Kitchen program in their working time) will need to be reassessed in subject populations from other employers and work settings. Second, as the current study had a short-term intervention and follow-up period, we could not evaluate the long-term efficacy of the intervention. Third, the questionnaires used in the study to examine the lifestyle behaviors, HR-QoL, and satisfaction were based on self-report. Although most of the questionnaires were highly validated and consistent with previous studies on Teaching Kitchen programs in Western countries (1, 3, 4, 31, 32), there may be self-reporting bias. Fourth, as a pilot study, the current study design had a small sample size with the lack of a control group. In addition, the participants of this study were predominantly male. However, the previous studies of lifestyle intervention for obesity in Japanese subjects showed that the effectiveness of such an approach was independent of gender (33, 34), which implies that the Japanese Teaching Kitchen is expected to be effective for women as well. Based on these limitations, the interpretation and generalizability of what we found in this study might be limited. As a next step, we will conduct a randomized controlled trial with a longer intervention and follow-up phase, with an adequate sample size of participants from diverse backgrounds. Such a follow-on trial will be necessary to establish scientific evidence in support of the long-term effectiveness of the Japanese Teaching Kitchen program, leading to further research development and social implementation of this novel approach in Japan and other Asian countries.

## 5 Conclusions

The current study showed that the Japanese Teaching Kitchen program used in this pilot study is feasible and may positively impact the disease risk profiles of Japanese adults with obesity, high blood pressure, and other chronic diseases associated with sub-optimal diets and lifestyles. These findings from an initial feasibility study of a teaching kitchen curriculum in Japan suggest that future studies in this area are worth pursuing to address obesity and obesity-related diseases.

## Data availability statement

The raw data supporting the conclusions of this article will be made available by the authors, without undue reservation.

## Ethics statement

The studies involving humans were approved by the Institutional Ethics Review Board of Osaka University Hospital. The studies were conducted in accordance with the local legislation and institutional requirements. The participants provided their written informed consent to participate in this study.

## Author contributions

MB: Conceptualization, Funding acquisition, Investigation, Methodology, Project administration, Resources, Visualization, Writing—original draft. SK: Data curation, Investigation, Resources, Writing—review & editing. AN: Investigation, Writing—review & editing. TH: Investigation, Writing—review & editing. HO: Data curation, Investigation, Resources, Writing—review & editing. CI: Writing—review & editing. YH: Writing—review & editing. YukF: Writing—review & editing. YuyF: Investigation, Writing—review & editing. HN: Investigation, Writing—review & editing. JK: Writing—review & editing. IM: Data curation, Formal analysis, Software, Writing—review & editing. YusF: Investigation, Visualization, Writing— review & editing. AY: Investigation, Writing—review & editing. TS: Writing—review & editing. TK: Writing—review & editing. IS: Funding acquisition, Supervision, Writing—review & editing. DE: Conceptualization, Methodology, Supervision, Writing—review & editing.
