# Erratum: Feasibility pilot study of a Japanese teaching kitchen program

**DOI:** 10.3389/fpubh.2024.1425324

**Published:** 2024-05-08

**Authors:** Frontiers Production Office

**Affiliations:** Frontiers Media SA, Lausanne, Switzerland

**Keywords:** teaching kitchen, diet, obesity, lifestyle modification, behavior modification program

Due to a production error, an incorrect Funding statement was provided:

“The authors declare that this study received funding from Cancerscan Inc. and Kubara Honke Group Co., Ltd. The funders had the following involvement in the study: creation of the web application (Cancerscan Inc.) and support of the culinary component (Kubara Honke Group Co., Ltd.). The funders were not involved in the study design, analysis and interpretation of data, or the decision to submit it for publication.”

The correct Funding statement is:

“The authors declare financial support was received for the research, authorship, and/or publication of this article. This research was funded by the Japan Diabetes Society, a junior scientist development grant, the G-7 Scholarship Foundation, a research and development grant, and Lotte Foundation, a Lotte Research Promotion Grant. This study also received funding from Cancerscan Inc., and Kubara Honke Group Co., Ltd. The funders were not involved in the study design, analysis and interpretation of data, or the decision to submit it for publication.”

Due to the above error, the Conflict of Interest statement was also incorrect. It originally stated:

“MB and HO are members of the Department of Lifestyle Medicine, a sponsored course endowed by Kubara Honke Group Co., Ltd. YusF and AY were employed by Cancerscan, Inc., Tokyo, Japan. TS and TK were employed by Kubara Honke Group Co., Ltd., Fukuoka, Japan.

The remaining authors declare that the research was conducted in the absence of any commercial or financial relationships that could be construed as a potential conflict of interest.”

The correct Conflict of Interest statement is:

“MB and HO are members of the Department of Lifestyle Medicine, a sponsored course endowed by Kubara Honke Group Co., Ltd. YusF and AY were employed by the Cancerscan, Inc, Tokyo, Japan. TS and TK were employed by Kubara Honke Group Co., Ltd, Fukuoka, Japan.

This study received funding from Cancerscan Inc and Kubara Honke Group Co., Ltd. The funders had the following involvement in the study: creation of the web application (Cancerscan Inc) and support of the culinary component (Kubara Honke Group Co., Ltd).

The remaining authors declare that the research was conducted in the absence of any commercial or financial relationships that could be construed as a potential conflict of interest.”

The publisher apologizes for this mistake. The original article has been updated.

