# Supplementary material for: Feasibility pilot study of a Japanese teaching kitchen program
Source: Front Public Health. 2023 Dec 7;11:1258434. doi: 10.3389/fpubh.2023.1258434 (PMC10749421; doi:10.3389/fpubh.2023.1258434)
Supplement: Supplementary file 1 [file Table_1.docx]

***Supplementary Material***

**Table of Contents:**

**Supplemental Table 1.** Baseline characteristics of participants at the time of entry in weekly group and bi-weekly group

**Supplemental Table 2.** An example of the timetable for a face-to-face class

**Supplemental Table 3.** Instruments for the assessment to be used in the study

**Supplemental Table 4.** Changes in biometrics at baseline, when finished the program, and one month later

**Supplemental Table 5.** Percent changes in biometrics at baseline and post-intervention

**Supplemental Table 6.** Percent changes in biometrics at baseline, when finished the program, and one month later

**Supplemental Table 7.** Percent changes in energy, nutrient and food intakes at baseline and post-intervention

**Supplemental Table 8.** Percent changes in physical activity and Athens insomnia scale at baseline and post-intervention

**Supplemental Table 9.** Physical activity and sleep duration during the program evaluated by smart bands

**Supplemental Table 10.** Changes in health-related quality of life (HR-QoL) at baseline and post-intervention

**Supplemental Table 11.** Percent changes in health-related quality of life (HR-QoL) at baseline and post-intervention

**Supplemental Table 1. Baseline characteristics of participants at the time of entry in weekly group and bi-weekly group**

|  | Weekly group | Bi-weekly group | p |
| --- | --- | --- | --- |
| *n* | 12 | 12 |  |
| Age, year | 38 [35, 51] | 39 [36, 43] | 0.31 |
| Sex (male/female) | 11/1 | 11/1 | 1.00 |
| Office (main office/research center), % | 75.0/25.0 | 75.0/25.0 | 1.00 |
| Height, cm | 171.6 ± 8.0 | 173.5 ± 6.5 | 0.53 |
| Weight, kg | 79.9 ± 9.3 | 81.7 ± 12.7 | 0.71 |
| BMI, kg/m^2^ | 27.1 ± 1.8 | 27.1 ± 3.9 | 0.96 |

Values are means ± standard deviations (SDs) for continuous variables. The differences between weekly and bi-weekly groups were tested using t-tests for continuous variables other than age, the Willcoxon rank test for age, and Fisher’s exact test for categorical variables. p<0.05 indicates statistically significant. BMI, body mass index; *n*, the number of participants.

**Supplemental Table 2. An example of the timetable for a face-to-face class**

| Contents | Contents | Time (min) |
| --- | --- | --- |
| Lectures on Nutrition | Lectures on nutritional evidence and practical skills to improve their diet | 15 |
| Lectures on Exercise/Mindfulness | Lectures on epidemiological evidence on exercise and/or mindfulness and suggestions to practice exercise and mindfulness in daily lives | 10 |
| Cooking | Hands-on culinary lessons to create the healthy recipes with chefs | 70 |
| Tasting | Eat the healthy and delicious foods that participants cooked by themselves | 15 |
| Wrap-up | Look back what the participants learned and experienced in the class | 10 |

**Supplemental Table 3. Instruments for the assessment to be used in the study**

| Assessments | Instruments | References |
| --- | --- | --- |
| Body composition | Body composition analyzer MC-780A-N (TANITA Co. Ltd, Tokyo, Japan) | https://api-img.tanita.co.jp/v=1666613921/files/user/support/catalog/mc780an.pdf?_ga=2.131902479.1446658362.1680500129-1432775949.1653973749 |
| Blood pressure | Digital sphygmomanometer (TERUMO, Tokyo, Japan) | https://www.terumo.co.jp/medical/equipment/me89.html |
| Dietary intakes | Self-administered long-food frequency questionnaire (Education Software Co., Ltd, Tokyo, Japan) | J. Epidemiol. 16:107-16, 2006 doi:10.2188/jea.16.107.  https://www.kyoikusw.co.jp/food-frequency-method/ |
| Eating behavior | Questionnaire of the guideline for obesity (The Japan Society for the Study of Obesity, Kyoto, Japan) | Cardiovasc. Diabetol. 11:107, 2012, doi:10.1186/1475-2840-11-107.  Cardiovasc. Diabetol. 14:8, 2015 doi:10.1186/s12933-015-0174-7.  Nutrients. 15:353, 2023 |
| Physical activity (self-administered) | International Physical Activity Questionnaire (Tokyo Medical University) | Med Sci Sports Exerc. 35:1381-95, 2003. |
| Sleep difficulty | Athens Insomnia Scale (Ministry of Health, Labour and Welfare, Japan) | Psychiatry Clin Neurosci 67: 420-5, 2013 |
| Health related quality of life | Medical Outcomes Study 36-Item Short-Form Health Survey, version2 (Qualitest, Kyoto, Japan) | J Clin Epidemiol 51: 1037-44, 1998  https://www.qualitest.jp/index.html |
| Physical activity and sleep duration | Smart band (Mi Band 6, Xiaomi, China) | <https://www.mi.com/jp/product/mi-smart-band-6/specs> |

**Supplemental Table 4. Changes in biometrics at baseline, when finished the program, and one month later**

|  | Baseline | Post-intervention | | Mean change | | p for trend | p for interaction |
| --- | --- | --- | --- | --- | --- | --- | --- |
|  |  | Finished | A month later | Finished | A month later |  |  |
| Weight, kg |  |  |  |  |  |  |  |
| Weekly (n=8) | 82.6 ± 3.5 | 81.4 ± 3.5 | 81.6 ± 3.5 | -1.2 ± 0.3 | -1.0 ± 0.5 | <0.001 |  |
| Bi-weekly (n=6) | 83.6 ± 6.4 | 81.5 ± 5.8 | 81.6 ± 6.0 | -2.2 ± 0.8 | -2.0 ± 0.9 | 0.001 |  |
| Total (n=14^a^) | 83.0 ± 3.3 | 81.4 ± 3.0 | 81.6 ± 3.1 | -1.6 ± 0.4 | -1.4 ± 0.5 | <0.001 | 0.40 |
| BMI, kg/m^2^ |  |  |  |  |  |  |  |
| Weekly (n=8) | 27.4 ± 0.8 | 27.0 ± 0.8 | 27.1 ± 0.8 | -0.4 ± 0.1 | -0.3 ± 0.2 | <0.001 |  |
| Bi-weekly (n=6) | 27.6 ± 2.0 | 26.9 ± 1.8 | 26.7 ± 2.0 | -0.7 ± 0.3 | -0.9 ± 0.3 | <0.001 |  |
| Total (n=14) | 27.5 ± 0.9 | 27.0 ± 0.9 | 26.9 ± 0.9 | -0.5 ± 0.1 | -0.6 ± 0.2 | <0.001 | 0.12 |
| SBP, mmHg |  |  |  |  |  |  |  |
| Weekly (n=8) | 133 ± 3 | 130 ± 7 | 130 ± 5 | -3 ± 5 | -2 ± 5 | 0.40 |  |
| Bi-weekly (n=6) | 137 ± 7 | 125 ± 4 | 126 ± 4 | -13 ± 6 | -11 ± 6 | 0.004 |  |
| Total (n=14) | 135 ± 4 | 128 ± 4 | 128 ± 4 | -7 ± 4 | -6 ± 4 | 0.008 | 0.26 |
| DBP, mmHg |  |  |  |  |  |  |  |
| Weekly (n=8) | 87 ± 3 | 82 ± 4 | 85 ± 4 | -4 ± 3 | -1 ± 4 | 0.26 |  |
| Bi-weekly (n=6) | 85 ± 4 | 82 ± 3 | 81 ± 3 | -4 ± 4 | -4 ± 2 | 0.03 |  |
| Total (n=14) | 86 ± 2 | 82 ± 2 | 84 ± 3 | -4 ± 2 | -3 ± 2 | 0.03 | 0.80 |
| Body fat mass, kg |  |  |  |  |  |  |  |
| Weekly (n=8) | 24.0 ± 2.1 | 23.1 ± 2.1 | 23.4 ± 2.0 | -0.9 ± 0.3 | -0.6 ± 0.3 | 0.001 |  |
| Bi-weekly (n=6) | 24.7 ± 4.0 | 22.6 ± 3.2 | 21.9 ± 3.2 | -2.1 ± 1.0 | -2.8 ± 1.2 | <0.001 |  |
| Total (n=14) | 24.3 ± 2.0 | 22.9 ± 1.7 | 22.8 ± 1.7 | -1.4 ± 0.5 | -1.5 ± 0.6 | <0.001 | 0.05 |
| Lean mass, kg |  |  |  |  |  |  |  |
| Weekly (n=8) | 55.5 ± 3.1 | 55.3 ± 3.0 | 55.1 ± 2.9 | -0.2 ± 0.4 | -0.4 ± 0.4 | 0.16 |  |
| Bi-weekly (n=6) | 55.9 ± 2.6 | 55.8 ± 2.6 | 56.6 ± 2.8 | -0.1 ± 0.8 | 0.8 ± 0.8 | 0.29 |  |
| Total (n=14) | 55.7 ± 2.0 | 55.5 ± 2.0 | 55.8 ± 2.0 | -0.2 ± 0.4 | 0.1 ± 0.4 | 0.98 | 0.14 |

Values are means ± standard errors ± SEs. **^a^**n=14 instead of 20 because measurements were not available for six participants because of work commitments. The tendency was tested using linear mixed effect models. p<0.05 indicates statistically significant. BMI, body mass index; SBP, systolic blood pressure; DBP, diastolic blood pressure.

**Supplemental Table 5. Percent changes in biometrics at baseline and post-intervention**

|  | Baseline | Post-intervention | %change | p | p for interaction |
| --- | --- | --- | --- | --- | --- |
| Weight, kg |  |  |  |  |  |
| Weekly (n=10) | 81.2 ± 3.3 | 80.0 ± 3.4 | -1.5 ± 0.4 | <0.001 |  |
| Bi-weekly (n=7) | 84.1 ± 5.5 | 82.4 ± 5.0 | -2.0 ± 0.7 | 0.02 |  |
| Total (n=17^a^) | 82.4 ± 2.9 | 80.9 ± 2.8 | -1.7 ± 0.4 | <0.001 | 0.56 |
| BMI, kg/m^2^ |  |  |  |  |  |
| Weekly (n=10) | 27.3 ± 0.8 | 26.9 (0.8) | -1.5 ± 0.4 | <0.001 |  |
| Bi-weekly (n=7) | 27.6 ± 1.7 | 27.0 (1.5) | -2.0 ± 0.7 | 0.02 |  |
| Total (n=17) | 27.4 ± 0.8 | 26.9 ± 0.8 | -1.7 ± 0.4 | <0.001 | 0.56 |
| SBP, mmHg |  |  |  |  |  |
| Weekly (n=10) | 134 ± 3 | 132 ± 6 | -2 ± 3 | 0.50 |  |
| Bi-weekly (n=7) | 140 ± 7 | 127 ± 4 | -8 ± 4 | 0.04 |  |
| Total (n=17) | 136 ± 4 | 130 ± 4 | -5 ± 2 | 0.05 | 0.15 |
| DBP, mmHg |  |  |  |  |  |
| Weekly (n=10) | 88 ± 2 | 83 ± 3 | -6 ± 2 | 0.02 |  |
| Bi-weekly (n=7) | 87 ± 4 | 84 ± 4 | -2 ± 4 | 0.51 |  |
| Total (n=17) | 87 ± 2 | 83 ± 2 | -4 ± 2 | 0.03 | 0.41 |
| Body fat mass, kg |  |  |  |  |  |
| Weekly (n=10) | 22.5 ± 2.2 | 21.5 ± 2.2 | -5.5 ± 2.0 | 0.01 |  |
| Bi-weekly (n=7) | 25.0 ± 3.4 | 22.9 ± 2.7 | -7.0 ± 2.7 | 0.02 |  |
| Total (n=17) | 23.5 ± 1.8 | 22.1 ± 1.7 | -6.1 ± 1.6 | <0.001 | 0.65 |
| Lean mass, kg |  |  |  |  |  |
| Weekly (n=10) | 55.6 ± 2.5 | 55.5 ± 2.5 | -0.3 ± 0.6 | 0.69 |  |
| Bi-weekly (n=7) | 56.1 ± 2.2 | 56.3 ± 2.2 | 0.6 ± 1.4 | 0.70 |  |
| Total (n=17) | 55.8 ± 1.7 | 55.8 ± 1.7 | 0.1 ± 0.7 | 0.90 | 0.56 |

Values are means ± standard errors (SEs). **^a^**n=17 instead of 20 because measurements were not available for three participants because of work commitments. The baseline and post-intervention differences were tested using linear mixed effect models. P<0.05 indicates statistically significant. BMI, body mass index; SBP, systolic blood pressure; DBP, diastolic blood pressure.

**Supplemental Table 6. Percent changes in biometrics at baseline, when finished the program, and one month later**

|  | Baseline | Post-intervention | | %change | | p for trend | p for interaction |
| --- | --- | --- | --- | --- | --- | --- | --- |
|  |  | Finished | A month later | Finished | A month later |  |  |
| Weight, kg |  |  |  |  |  |  |  |
| Weekly (n=8) | 82.6 ± 3.5 | 81.4 ± 3.5 | 81.6 ± 3.5 | -1.4 ± 0.4 | -1.2 ± 0.5 | <0.001 |  |
| Bi-weekly (n=6) | 83.6 ± 6.4 | 81.5 ± 5.8 | 81.6 ± 6.0 | -2.4 ± 0.7 | -2.3 ± 0.8 | <0.001 |  |
| Total (n=14^a^) | 83.0 ± 3.3 | 81.4 ± 3.0 | 81.6 ± 3.1 | -1.8 ± 0.4 | -1.7 ± 0.5 | <0.001 | 0.40 |
| BMI, kg/m^2^ |  |  |  |  |  |  |  |
| Weekly (n=8) | 27.4 ± 0.8 | 27.0 ± 0.8 | 27.1 ± 0.8 | -1.4 ± 0.4 | -1.2 ± 0.5 | <0.001 |  |
| Bi-weekly (n=6) | 27.6 ± 2.0 | 26.9 ± 1.8 | 26.7 ± 2.0 | -2.4 ± 0.7 | -3.1 ± 1.0 | <0.001 |  |
| Total (n=14) | 27.5 ± 0.9 | 27.0 ± 0.9 | 26.9 ± 0.9 | -1.8 ± 0.4 | -2.0 ± 0.6 | <0.001 | 0.10 |
| SBP, mmHg |  |  |  |  |  |  |  |
| Weekly (n=8) | 133 ± 3 | 130 ± 7 | 130 ± 5 | -2.3 ± 3.5 | -1.7 ± 3.4 | 0.35 |  |
| Bi-weekly (n=6) | 137 ± 7 | 125 ± 4 | 126 ± 4 | -8.3 ± 4.2 | -7.3 ± 4.3 | 0.008 |  |
| Total (n=14) | 135 ± 4 | 128 ± 4 | 128 ± 4 | -4.8 ± 2.7 | -4.1 ± 2.7 | 0.01 | 0.34 |
| DBP, mmHg |  |  |  |  |  |  |  |
| Weekly (n=8) | 87 ± 3 | 82 ± 4 | 85 ± 4 | -5.1 ± 2.9 | -1.1 ± 4.8 | 0.32 |  |
| Bi-weekly (n=6) | 85 ± 4 | 82 ± 3 | 81 ± 3 | -3.5 ± 3.9 | -4.4 ± 2.1 | 0.04 |  |
| Total (n=14) | 86 ± 2 | 82 ± 2 | 84 ± 3 | -4.4 ± 2.3 | -2.5 ± 2.8 | 0.049 | 0.78 |
| Body fat mass, kg |  |  |  |  |  |  |  |
| Weekly (n=8) | 24.0 ± 2.1 | 23.1 ± 2.1 | 23.4 ± 2.0 | -3.9 ± 1.3 | -2.3 ± 1.0 | 0.002 |  |
| Bi-weekly (n=6) | 24.7 ± 4.0 | 22.6 ± 3.2 | 21.9 ± 3.2 | -6.8 ± 3.2 | -10.5 ± 3.3 | <0.001 |  |
| Total (n=14) | 24.3 ± 2.0 | 22.9 ± 1.7 | 22.8 ± 1.7 | -5.2 ± 1.5 | -5.8 ± 1.8 | <0.001 | 0.03 |
| Lean mass, kg |  |  |  |  |  |  |  |
| Weekly (n=8) | 55.5 ± 3.1 | 55.3 ± 3.0 | 55.1 ± 2.9 | -0.4 ± 0.8 | -0.6 ± 0.7 | 0.26 |  |
| Bi-weekly (n=6) | 55.9 ± 2.6 | 55.8 ± 2.6 | 56.6 ± 2.8 | 0.0 ± 1.5 | 1.4 ± 1.5 | 0.32 |  |
| Total (n=14) | 55.7 ± 2.0 | 55.5 ± 2.0 | 55.8 ± 2.0 | -0.2 ± 0.8 | 0.2 ± 0.8 | 0.85 | 0.20 |

Values are means ± standard errors ± SEs. **^a^**n=14 instead of 20 because measurements were not available for six participants because of work commitments. The tendency was tested using linear mixed effect models. p<0.05 indicates statistically significant. BMI, body mass index; SBP, systolic blood pressure; DBP, diastolic blood pressure.

**Supplemental Table 7. Percent changes in energy, nutrient and food intakes at baseline and post-intervention**

|  | Baseline | Post-intervention | %change | p | P for interaction |
| --- | --- | --- | --- | --- | --- |
| Total energy intake, kcal |  |  |  |  |  |
| Weekly (n=10) | 2176 ± 71 | 2133 ± 63 | -1.7 ± 2.0 | 0.39 |  |
| Bi-weekly (n=7) | 2247 ± 55 | 2162 ± 40 | -3.6 ± 1.2 | 0.01 |  |
| Total (n=17) | 2205 ± 47 | 2145 ± 40 | -2.5 ± 1.3 | 0.06 | 0.52 |
| Protein, g |  |  |  |  |  |
| Weekly (n=10) | 86.3 ± 1.8 | 85.2 ± 1.9 | -1.3 ± 1.3 | 0.33 |  |
| Bi-weekly (n=7) | 88.9 ± 1.8 | 86.6 ± 0.9 | -2.5 ± 1.3 | 0.07 |  |
| Total (n=17) | 87.4 ± 1.2 | 85.8 ± 1.1 | -1.8 ± 0.9 | 0.05 | 0.50 |
| Total fat, g |  |  |  |  |  |
| Weekly (n=10) | 64.0 ± 1.1 | 63.1 ± 1.1 | -1.4 ± 1.3 | 0.3 |  |
| Bi-weekly (n=7) | 65.9 ± 0.8 | 63.8 ± 0.9 | -3.2 ± 1.1 | 0.01 |  |
| Total (n=17) | 64.8 ± 0.7 | 63.4 ± 0.7 | -2.2 ± 0.9 | 0.03 | 0.38 |
| Carbohydrate, g |  |  |  |  |  |
| Weekly (n=10) | 286.6 ± 14.8 | 283.2 ± 12.8 | -0.4 ± 3.3 | 0.75 |  |
| Bi-weekly (n=7) | 288.3 ± 11.3 | 282.0 ± 6.1 | -1.7 ± 2.2 | 0.34 |  |
| Total (n=17) | 287.3 ± 9.6 | 282.7 ± 7.8 | -1.0 ± 2.1 | 0.48 | 0.82 |
| Total dietary fiber, g |  |  |  |  |  |
| Weekly (n=10) | 17.9 ± 0.5 | 17.7 ± 0.5 | -0.8 ± 1.9 | 0.66 |  |
| Bi-weekly (n=7) | 19.2 ± 1.2 | 18.8 ± 0.9 | -1.4 ± 3.0 | 0.51 |  |
| Total (n=17) | 18.4 ± 0.6 | 18.2 ± 0.5 | -1.1 ± 1.6 | 0.41 | 0.73 |
| Salt, g |  |  |  |  |  |
| Weekly (n=10) | 10.7 ± 0.4 | 10.3 ± 0.4 | -3.4 ± 2.3 | 0.15 |  |
| Bi-weekly (n=7) | 11.7 ± 0.7 | 10.8 ± 0.4 | -6.9 ± 2.5 | 0.02 |  |
| Total (n=17) | 11.1 ± 0.4 | 10.5 ± 0.3 | -4.8 ± 1.7 | 0.008 | 0.24 |
| Vegetables, g |  |  |  |  |  |
| Weekly (n=10) | 338.6 ± 9.1 | 349.5 ± 15.5 | 3.6 ± 5.0 | 0.51 |  |
| Bi-weekly (n=7) | 390.7 ± 35.2 | 412.0 ± 24.6 | 8.6 ± 8.9 | 0.5 |  |
| Total (n=17) | 360.1 ± 16.1 | 375.2 ± 15.2 | 5.7 ± 4.6 | 0.33 | 0.75 |
| Fruits, g |  |  |  |  |  |
| Weekly (n=10) | 131.9 ± 4.8 | 129.3 ± 4.7 | -1.9 ± 1.0 | 0.09 |  |
| Bi-weekly (n=7) | 140.7 ± 14.6 | 150.6 ± 20.5 | 5.5 ± 3.7 | 0.17 |  |
| Total (n=17) | 135.5 ± 6.4 | 138.1 ± 8.9 | 1.1 ± 1.8 | 0.43 | 0.04 |
| Fish and shellfish, g |  |  |  |  |  |
| Weekly (n=10) | 101.1 ± 2.2 | 101.9 ± 3.2 | 0.8 ± 2.3 | 0.73 |  |
| Bi-weekly (n=7) | 106.1 ± 5.1 | 105.3 ± 3.7 | 0.4 ± 5.5 | 0.89 |  |
| Total (n=17) | 103.2 ± 2.5 | 103.3 ± 2.4 | 0.6 ± 2.5 | 0.97 | 0.78 |
| Meats, g |  |  |  |  |  |
| Weekly (n=10) | 73.8 ± 2.6 | 72.1 ± 4.8 | -1.6 ± 6.4 | 0.74 |  |
| Bi-weekly (n=7) | 79.3 ± 5.0 | 69.1 ± 4.9 | -12.3 ± 4.2 | 0.02 |  |
| Total (n=17) | 76.1 ± 2.5 | 70.9 ± 3.4 | -6.0 ± 4.3 | 0.14 | 0.22 |
| Eggs, g |  |  |  |  |  |
| Weekly (n=10) | 35.5 ± 0.4 | 34.9 ± 0.7 | -1.6 ± 1.9 | 0.38 |  |
| Bi-weekly (n=7) | 37.3 ± 1.7 | 36.7 ± 0.6 | -0.8 ± 2.8 | 0.66 |  |
| Total (n=17) | 36.2 ± 0.7 | 35.6 ± 0.5 | -1.3 ± 1.6 | 0.36 | 0.98 |
| Dairy, g |  |  |  |  |  |
| Weekly (n=10) | 194.1 ± 42.7 | 162.4 ± 22.2 | -3.5 ± 9.5 | 0.48 |  |
| Bi-weekly (n=7) | 195.7 ± 38.8 | 187.4 ± 17.1 | 8.3 ± 16.7 | 0.81 |  |
| Total (n=17) | 194.8 ± 28.9 | 172.7 ± 14.8 | 1.3 ± 8.7 | 0.45 | 0.70 |
| Alcoholic beverages, g |  |  |  |  |  |
| Weekly (n=10) | 8.9 ± 4.4 | 4.3 ± 1.7 | 33.3 ± 64.3 | 0.21 |  |
| Bi-weekly (n=7) | 11.2 ± 4.9 | 9.6 ± 4.2 | 76.1 ± 98.2 | 0.11 |  |
| Total (n=17) | 9.9 ± 3.2 | 6.5 ± 2.0 | 51.7 ± 53.8 | 0.12 | 0.64 |
| Confectionaries, g |  |  |  |  |  |
| Weekly (n=10) | 24.5 ± 5.2 | 19.2 ± 4.7 | 39.3 ± 59 | 0.16 |  |
| Bi-weekly (n=7) | 30.6 ± 7.3 | 21.7 ± 10.2 | -27.3 ± 18.5 | 0.24 |  |
| Total (n=17) | 27.0 ± 4.2 | 20.2 ± 4.8 | 11.9 ± 35.7 | 0.06 | 0.63 |

Values are means ± standard errors (SEs). The baseline and postintervention differences were tested using linear mixed effect models. P<0.05 indicates statistically significant.

**Supplemental Table 8. Percent changes in physical activity and Athens insomnia scale at baseline and post-intervention**

|  | Baseline | Post-intervention | %change | p | p for interaction |
| --- | --- | --- | --- | --- | --- |
| Physical activity, MET-h/week |  |  |  |  |  |
| Weekly (n=10) | 22.8 ± 5.0 | 27.4 ± 6.1 | 30.4 ± 16.3 | 0.08 |  |
| Bi-weekly (n=5) | 19.2 ± 5.1 | 22.0 ± 5.1 | 153.6 ± 140.2 | 0.30 |  |
| Total (n=15) | 21.6 ± 3.7 | 25.6 ± 4.3 | 71.5 ± 47.2 | 0.14 | 0.22 |
| Athens Insomnia Scale |  |  |  |  |  |
| Weekly (n=10) | 4.4 ± 0.6 | 3.4 ± 1.1 | -12.2 ± 23.8 | 0.62 |  |
| Bi-weekly (n=6) | 8.4 ± 1.9 | 7.0 ± 2.2 | -4.5 ± 22.5 | 0.85 |  |
| Total (n=16) | 5.8 ± 0.9 | 4.8 ± 1.1 | -9.3 ± 16.6 | 0.58 | 0.83 |

Values are means ± standard errors (SEs). Physical activities were assessed using international physical activity questionnaire (IPAQ). n=15 for physical activity and 16 for Athens insomnia scale instead of 17 because of missing values. The baseline and postintervention differences were tested using linear mixed effect models. p<0.05 indicates statistically significant. MET, metabolic equivalent.

**Supplemental Table 9. Physical activity and sleep duration during the program evaluated by smart bands**

|  | Total | Weekly group | Bi-weekly group | p for interaction |
| --- | --- | --- | --- | --- |
| n**^a^** | 13 | 7 | 6 |  |
| Physical activity, MET-h/week | 29.0 (4.4) | 36.4 (6.7) | 20.5 (3.1) | 0.07 |
| Sleep duration, h | 6.3 (0.3) | 6.7 (0.3) | 5.9 (0.4) | 0.20 |

Values are means ± standard errors (SEs). Physical activity and sleep duration were assessed using smart bands. **^a^**n=13 instead of 17 because two participants did not return the data and two participants had missing data. The differences between groups were tested using linear mixed effect models. p<0.05 indicates statistically significant differences. MET, metabolic equivalent; h, hour.

**Supplemental Table 10. Changes in health-related quality of life (HR-QoL) at baseline and post-intervention**

|  | Baseline | Post-intervention | Mean change | p | P for interaction |
| --- | --- | --- | --- | --- | --- |
| Physical functioning |  |  |  |  |  |
| Weekly (n=10) | 52.2 ± 1.0 | 52.5 ± 1.2 | 0.3 ± 1.2 | 0.83 |  |
| Bi-weekly (n=6) | 55.3 ± 0.9 | 55.3 ± 0.9 | 0.0 ± 1.2 | 1.00 |  |
| Total (n=16**^a^**) | 53.4 ± 0.8 | 53.5 ± 0.9 | 0.2 ± 0.8 | 0.85 | 0.89 |
| Role physical |  |  |  |  |  |
| Weekly (n=10) | 52.4 ± 2.1 | 53.0 ± 1.9 | 0.6 ± 1.3 | 0.68 |  |
| Bi-weekly (n=6) | 54.8 ± 1.4 | 53.3 ± 3.4 | -1.5 ± 1.2 | 0.5 |  |
| Total (n=16**^a^**) | 53.3 ± 1.4 | 53.1 ± 1.7 | -0.2 ± 1.1 | 0.87 | 0.40 |
| Bodily pain |  |  |  |  |  |
| Weekly (n=10) | 46.1 ± 2.4 | 52.7 ± 2.7 | 6.6 ± 2.7 | 0.03 |  |
| Bi-weekly (n=6) | 49.2 ± 3.6 | 51.6 ± 3.0 | 2.4 ± 3.7 | 0.53 |  |
| Total (n=16**^a^**) | 47.3 ± 2.0 | 52.3 ± 2.0 | 5.0 ± 2.2 | 0.03 | 0.37 |
| General health |  |  |  |  |  |
| Weekly (n=10) | 48.3 ± 1.9 | 53.2 ± 2.3 | 4.9 ± 2.1 | 0.03 |  |
| Bi-weekly (n=6) | 51.5 ± 2.9 | 54.0 ± 3.1 | 2.6 ± 1.2 | 0.05 |  |
| Total (n=16**^a^**) | 49.5 ± 1.6 | 53.5 ± 1.8 | 4.1 ± 1.4 | 0.006 | 0.41 |
| Vitality |  |  |  |  |  |
| Weekly (n=10) | 47.7 ± 2.4 | 52.0 ± 2.6 | 4.3 ± 1.8 | 0.03 |  |
| Bi-weekly (n=6) | 45.3 ± 4.0 | 48.8 ± 3.5 | 3.5 ± 4.4 | 0.44 |  |
| Total (n=16**^a^**) | 46.8 ± 2.1 | 50.8 ± 2.1 | 4.0 ± 1.9 | 0.047 | 0.86 |
| Social functioning |  |  |  |  |  |
| Weekly (n=10) | 51.5 ± 3.1 | 55.5 ± 1.2 | 3.9 ± 3.0 | 0.21 |  |
| Bi-weekly (n=6) | 54.9 ± 1.9 | 54.9 ± 1.9 | 0.0 ± 0.0 | 1 |  |
| Total (n=16**^a^**) | 52.8 ± 2.0 | 55.2 ± 1.0 | 2.5 ± 1.9 | 0.21 | 0.33 |
| Role emotional |  |  |  |  |  |
| Weekly (n=10) | 48.6 ± 2.6 | 51.2 ± 2.4 | 2.6 ± 2.0 | 0.21 |  |
| Bi-weekly (n=6) | 51.2 ± 3.0 | 54.9 ± 1.9 | 3.7 ± 2.4 | 0.15 |  |
| Total (n=16**^a^**) | 49.6 ± 1.9 | 52.6 ± 1.7 | 3.0 ± 1.5 | 0.05 | 0.73 |
| Mental health |  |  |  |  |  |
| Weekly (n=10) | 51.0 ± 2.2 | 52.0 ± 1.8 | 1.0 ± 1.3 | 0.44 |  |
| Bi-weekly (n=6) | 50.8 ± 4.5 | 51.6 ± 4.3 | 0.8 ± 3.5 | 0.82 |  |
| Total (n=16**^a^**) | 50.9 ± 2.1 | 51.8 ± 1.9 | 0.9 ± 1.5 | 0.53 | 0.96 |
| Physical component score |  |  |  |  |  |
| Weekly (n=10) | 49.6 ± 1.8 | 51.9 ± 1.3 | 2.3 ± 1.5 | 0.15 |  |
| Bi-weekly (n=6) | 53.4 ± 2.4 | 53.8 ± 3.4 | 0.5 ± 2.8 | 0.88 |  |
| Total (n=16**^a^**) | 51.0 ± 1.5 | 52.6 ± 1.5 | 1.6 ± 1.4 | 0.26 | 0.53 |
| Mental component score |  |  |  |  |  |
| Weekly (n=10) | 47.5 ± 2.4 | 52.2 ± 2.2 | 4.7 ± 1.8 | 0.02 |  |
| Bi-weekly (n=6) | 46.3 ± 2.7 | 49.3 ± 2.3 | 3.1 ± 3.2 | 0.36 |  |
| Total (n=16**^a^**) | 47.0 ± 1.8 | 51.1 ± 1.6 | 4.1 ± 1.6 | 0.01 | 0.62 |

Values are means ± standard errors (SEs). **^a^**n=16 instead of 17 because of the missing value. The baseline and postintervention differences were tested using linear mixed effect models. p<0.05 indicates statistically significant.

**Supplemental Table 11. Percent changes in health-related quality of life (HR-QoL) at baseline and post-intervention**

|  | Baseline | Post-intervention | %change | p | P for interaction |
| --- | --- | --- | --- | --- | --- |
| Physical functioning |  |  |  |  |  |
| Weekly (n=10) | 52.2 ± 1.0 | 52.5 ± 1.2 | 0.7 ± 2.3 | 0.77 |  |
| Bi-weekly (n=6) | 55.3 ± 0.9 | 55.3 ± 0.9 | 0.1 ± 2.2 | 0.96 |  |
| Total (n=16**^a^**) | 53.4 ± 0.8 | 53.5 ± 0.9 | 0.5 ± 1.6 | 0.77 | 0.87 |
| Role physical |  |  |  |  |  |
| Weekly (n=10) | 52.4 ± 2.1 | 53.0 ± 1.9 | 1.7 ± 3.3 | 0.62 |  |
| Bi-weekly (n=6) | 54.8 ± 1.4 | 53.3 ± 3.4 | -3.1 ± 4.3 | 0.48 |  |
| Total (n=16**^a^**) | 53.3 ± 1.4 | 53.1 ± 1.7 | -0.1 ± 2.6 | 0.96 | 0.38 |
| Bodily pain |  |  |  |  |  |
| Weekly (n=10) | 46.1 ± 2.4 | 52.7 ± 2.7 | 15.6 ± 5.8 | 0.02 |  |
| Bi-weekly (n=6) | 49.2 ± 3.6 | 51.6 ± 3.0 | 6.9 ± 8.2 | 0.42 |  |
| Total (n=16**^a^**) | 47.3 ± 2.0 | 52.3 ± 2.0 | 12.3 ± 4.7 | 0.01 | 0.38 |
| General health |  |  |  |  |  |
| Weekly (n=10) | 48.3 ± 1.9 | 53.2 ± 2.3 | 11.0 ± 4.9 | 0.04 |  |
| Bi-weekly (n=6) | 51.5 ± 2.9 | 54.0 ± 3.1 | 5.1 ± 2.5 | 0.07 |  |
| Total (n=16**^a^**) | 49.5 ± 1.6 | 53.5 ± 1.8 | 8.8 ± 3.2 | 0.01 | 0.39 |
| Vitality |  |  |  |  |  |
| Weekly (n=10) | 47.7 ± 2.4 | 52.0 ± 2.6 | 9.6 ± 3.6 | 0.01 |  |
| Bi-weekly (n=6) | 45.3 ± 4.0 | 48.8 ± 3.5 | 11.1 ± 10.7 | 0.33 |  |
| Total (n=16**^a^**) | 46.8 ± 2.1 | 50.8 ± 2.1 | 10.2 ± 4.4 | 0.03 | 0.88 |
| Social functioning |  |  |  |  |  |
| Weekly (n=10) | 51.5 ± 3.1 | 55.5 ± 1.2 | 12.4 ± 9.7 | 0.22 |  |
| Bi-weekly (n=6) | 54.9 ± 1.9 | 54.9 ± 1.9 | 0.0 ± 0.0 | 1.00 |  |
| Total (n=16**^a^**) | 52.8 ± 2.0 | 55.2 ± 1.0 | 7.7 ± 6.1 | 0.22 | 0.34 |
| Role emotional |  |  |  |  |  |
| Weekly (n=10) | 48.6 ± 2.6 | 51.2 ± 2.4 | 6.7 ± 5.3 | 0.22 |  |
| Bi-weekly (n=6) | 51.2 ± 3.0 | 54.9 ± 1.9 | 8.6 ± 5.6 | 0.16 |  |
| Total (n=16**^a^**) | 49.6 ± 1.9 | 52.6 ± 1.7 | 7.4 ± 3.8 | 0.06 | 0.82 |
| Mental health |  |  |  |  |  |
| Weekly (n=10) | 51.0 ± 2.2 | 52.0 ± 1.8 | 2.6 ± 2.6 | 0.33 |  |
| Bi-weekly (n=6) | 50.8 ± 4.5 | 51.6 ± 4.3 | 3.8 ± 8.1 | 0.65 |  |
| Total (n=16**^a^**) | 50.9 ± 2.1 | 51.8 ± 1.9 | 3.0 ± 3.3 | 0.36 | 0.86 |
| Physical component score |  |  |  |  |  |
| Weekly (n=10) | 49.6 ± 1.8 | 51.9 ± 1.3 | 5.4 ± 3.1 | 0.10 |  |
| Bi-weekly (n=6) | 53.4 ± 2.4 | 53.8 ± 3.4 | 1.0 ± 5.3 | 0.85 |  |
| Total (n=16**^a^**) | 51.0 ± 1.5 | 52.6 ± 1.5 | 3.8 ± 2.7 | 0.18 | 0.45 |
| Mental component score |  |  |  |  |  |
| Weekly (n=10) | 47.5 ± 2.4 | 52.2 ± 2.2 | 11.3 ± 4.6 | 0.03 |  |
| Bi-weekly (n=6) | 46.3 ± 2.7 | 49.3 ± 2.3 | 8.4 ± 8.3 | 0.34 |  |
| Total (n=16**^a^**) | 47.0 ± 1.8 | 51.1 ± 1.6 | 10.2 ± 4.1 | 0.02 | 0.75 |

Values are means ± standard errors (SEs). **^a^**n=16 instead of 17 because of the missing value. The baseline and postintervention differences were tested using linear mixed effect models. p<0.05 indicates statistically significant.
